# Supplementary material for: The causal relationship between the human gut microbiota and pyogenic arthritis: a Mendelian randomization study
Source: Front Cell Infect Microbiol. 2024 Nov 26;14:1452480. doi: 10.3389/fcimb.2024.1452480 (PMC11629706; doi:10.3389/fcimb.2024.1452480)
Supplement: Supplementary file 1 [file DataSheet1.zip › mendelian test/ebi-a-GCST90027711.csv_forest.pdf]

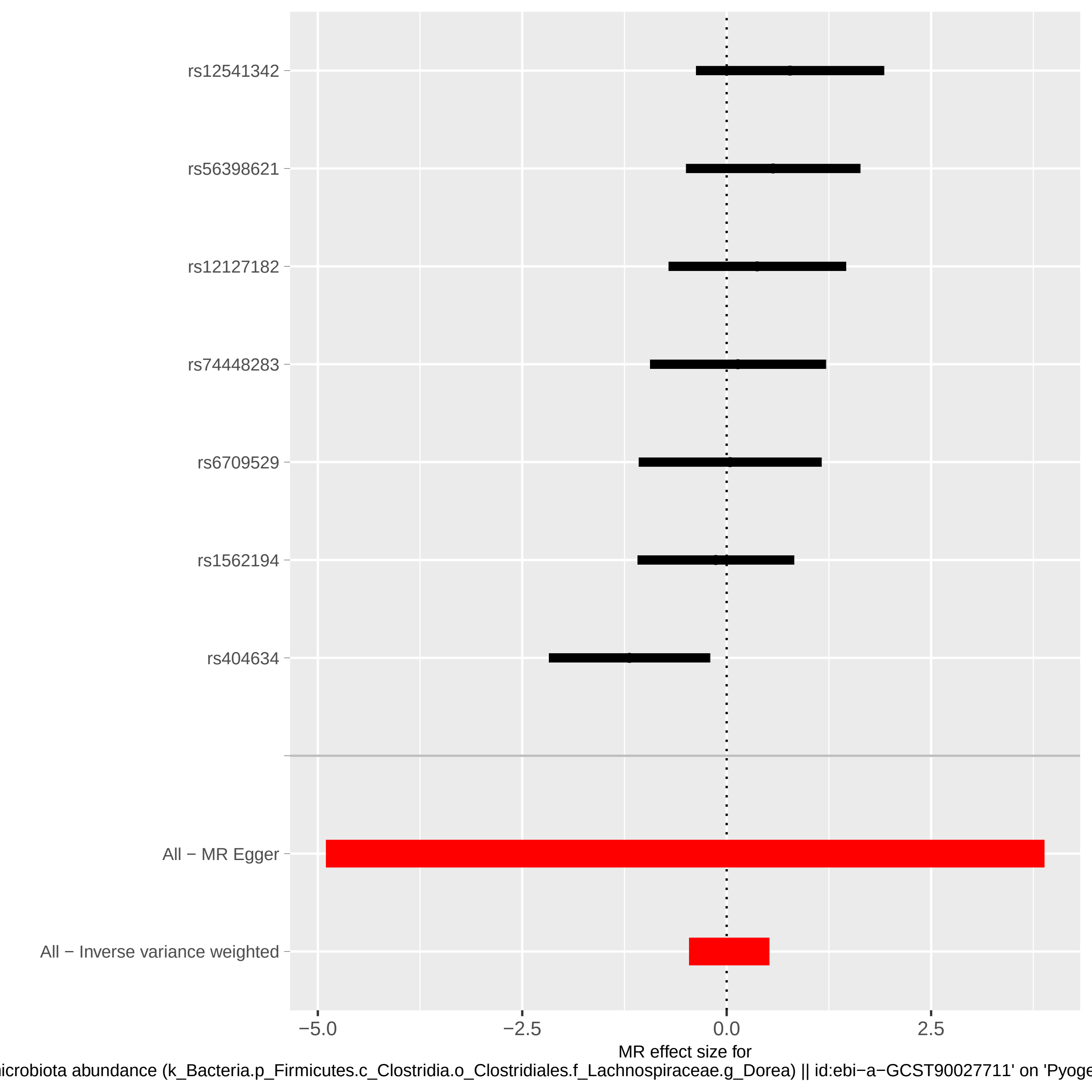

rs12541342
rs56398621
rs12127182
rs74448283
rs6709529
rs1562194
rs404634
All − MR Egger
All − Inverse variance weighted
−5.0
−2.5
0.0
2.5
MR effect size for
icrobiota abundance (k_Bacteria.p_Firmicutes.c_Clostridia.o_Clostridiales.f_Lachnospiraceae.g_Dorea) || id:ebi−a−GCST90027711' on 'Pyoge
